# Supplementary figures and images for: Visual Processing During the Interictal Period Between Migraines: A Meta-Analysis
Source: Neuropsychol Rev. 2022 Sep 17;33(4):765–82. doi: 10.1007/s11065-022-09562-3 (PMC10770263; doi:10.1007/s11065-022-09562-3)

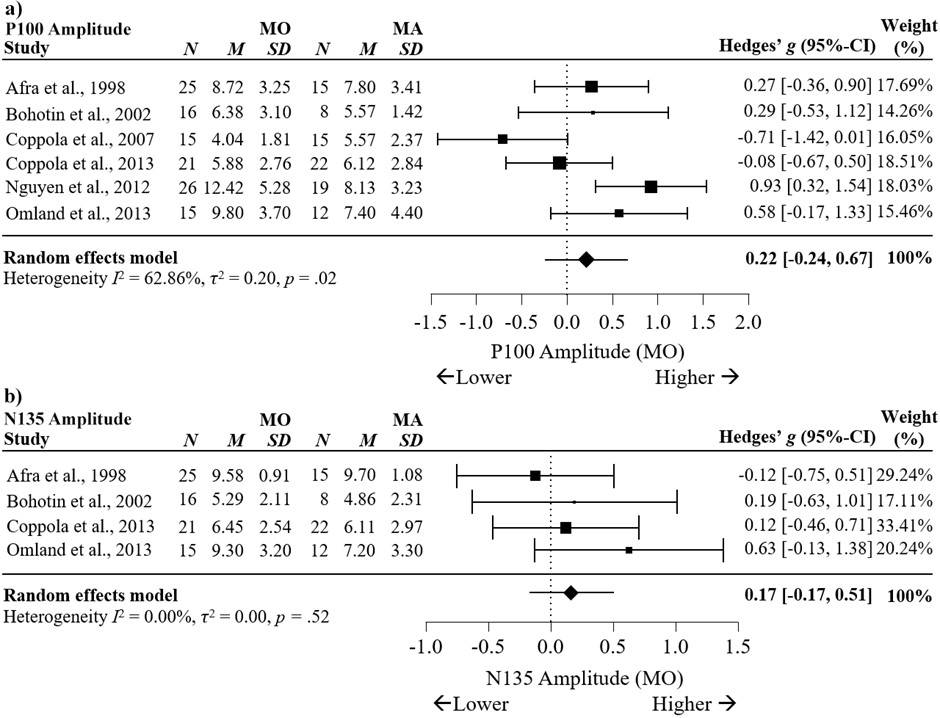

Supplement: Supplementary file 1 — Supplementary file1 (DOCX 160 KB) [file 11065_2022_9562_MOESM1_ESM.docx]

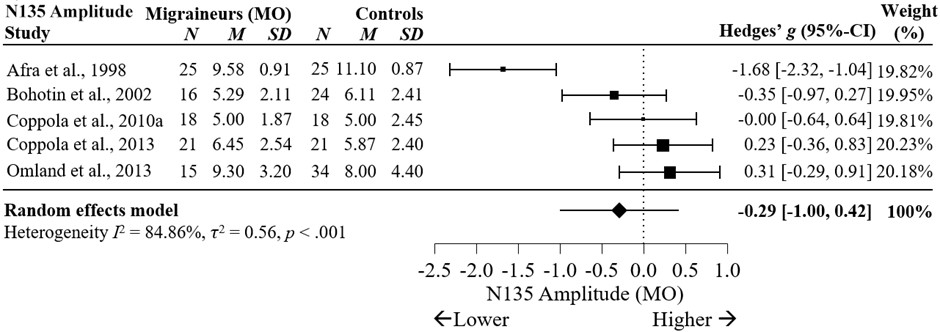

Supplement: Supplementary file 2 — Supplementary file2 (DOCX 87 KB) [file 11065_2022_9562_MOESM2_ESM.docx]

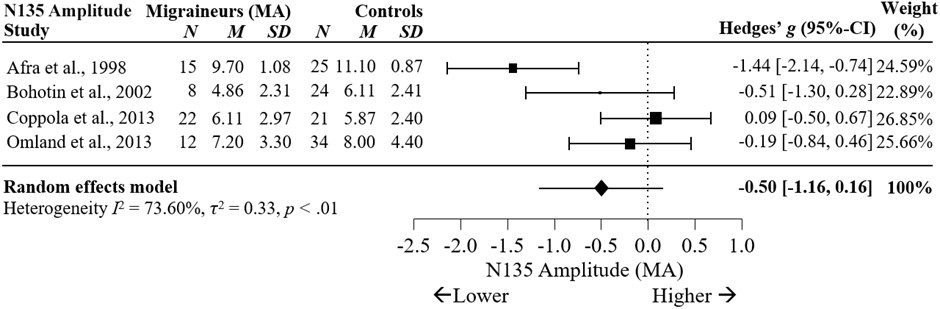

Supplement: Supplementary file 3 — Supplementary file3 (DOCX 83 KB) [file 11065_2022_9562_MOESM3_ESM.docx]

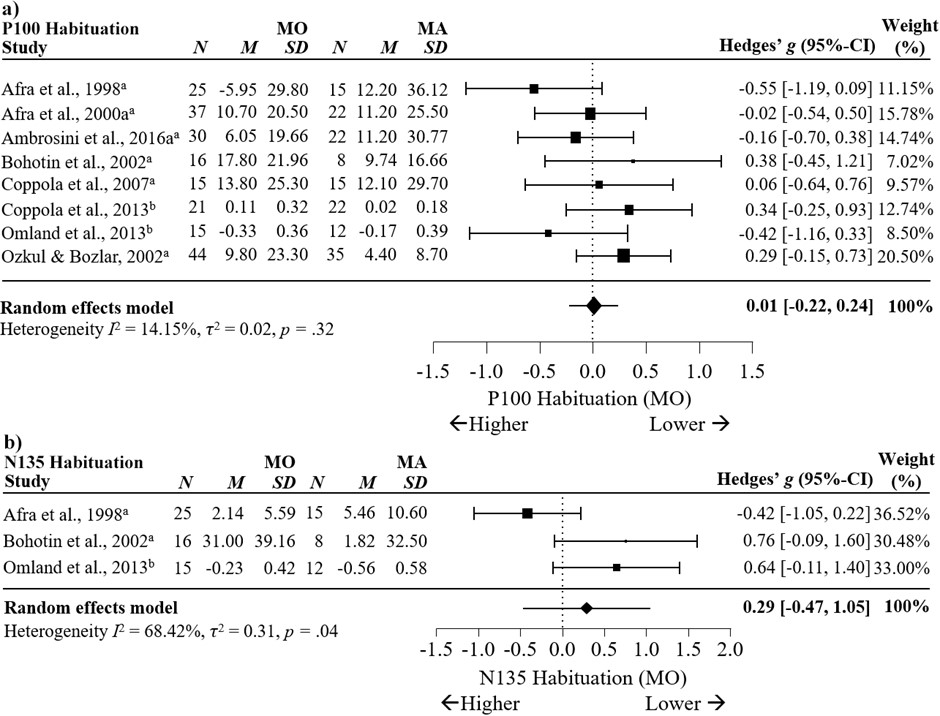

Supplement: Supplementary file 4 — Supplementary file4 (DOCX 164 KB) [file 11065_2022_9562_MOESM4_ESM.docx]

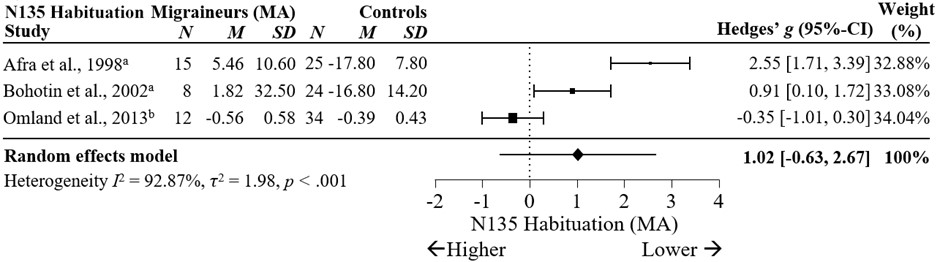

Supplement: Supplementary file 5 — Supplementary file5 (DOCX 71 KB) [file 11065_2022_9562_MOESM5_ESM.docx]

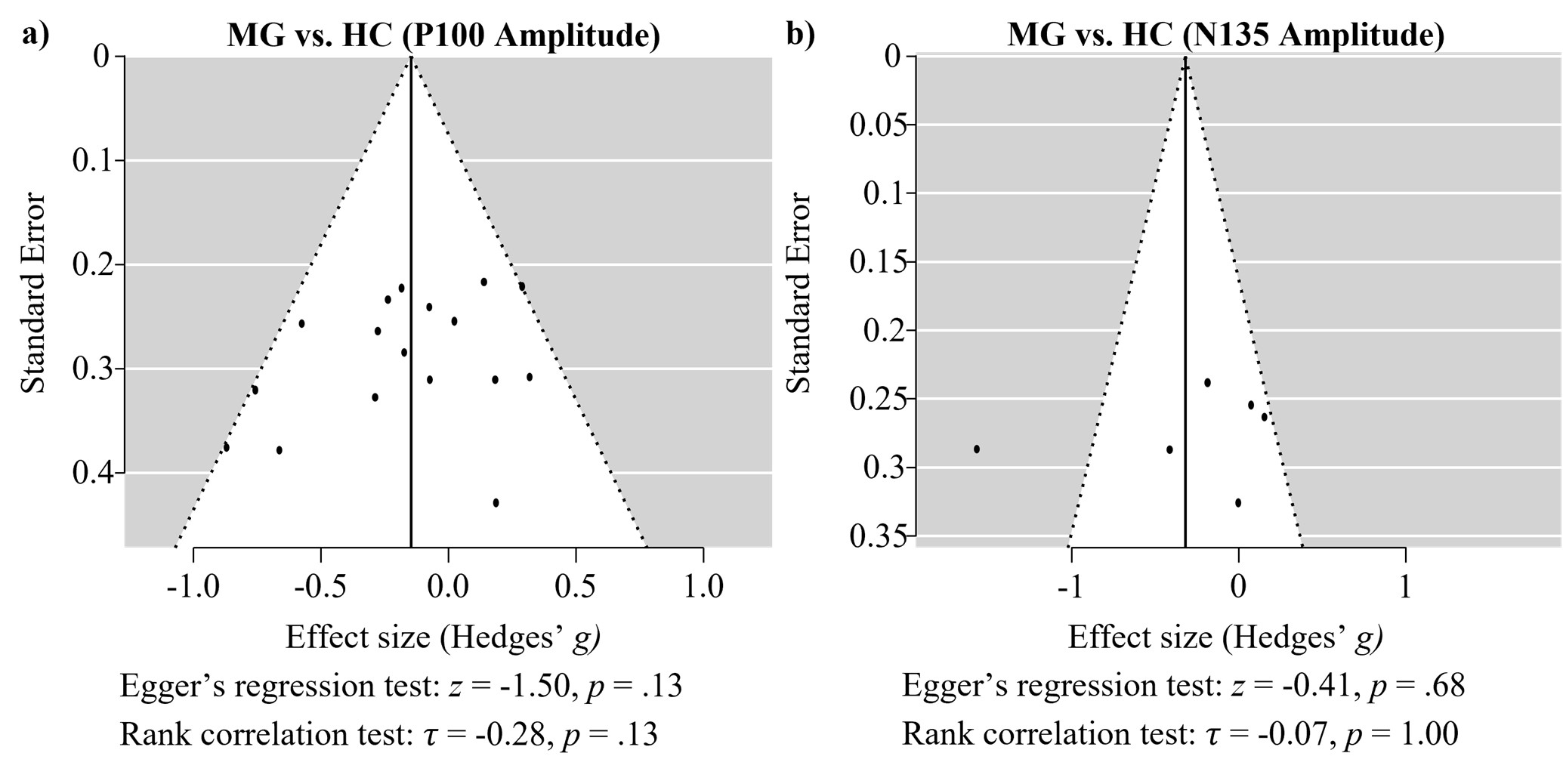

Supplement: Supplementary file 6 — Supplementary file6 (DOCX 218 KB) [file 11065_2022_9562_MOESM6_ESM.docx]

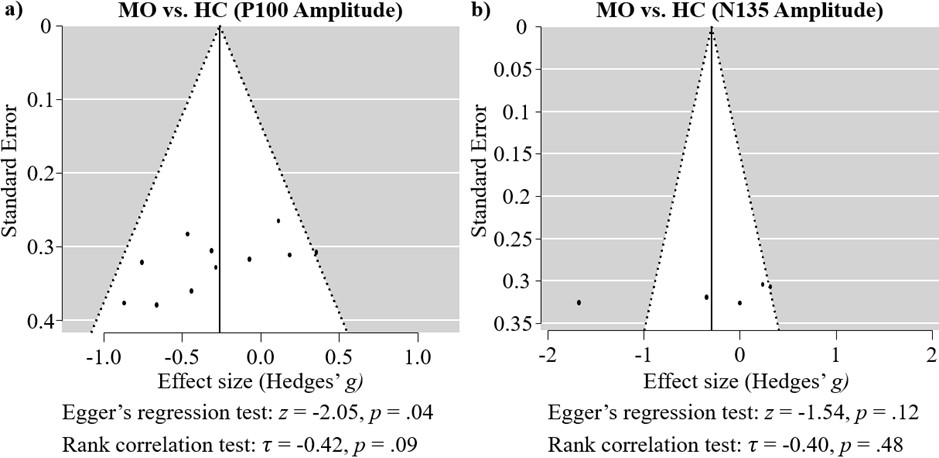

Supplement: Supplementary file 7 — Supplementary file7 (DOCX 86 KB) [file 11065_2022_9562_MOESM7_ESM.docx]

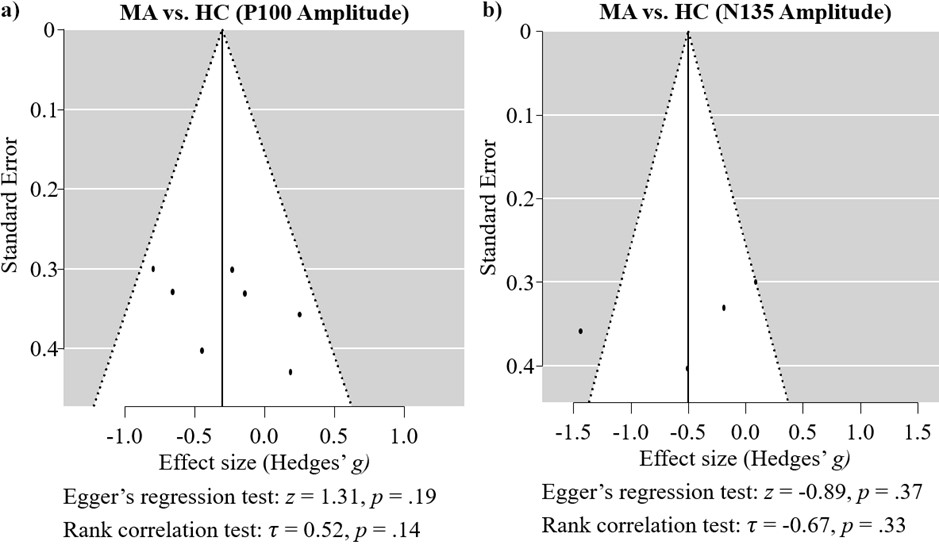

Supplement: Supplementary file 8 — Supplementary file8 (DOCX 88 KB) [file 11065_2022_9562_MOESM8_ESM.docx]

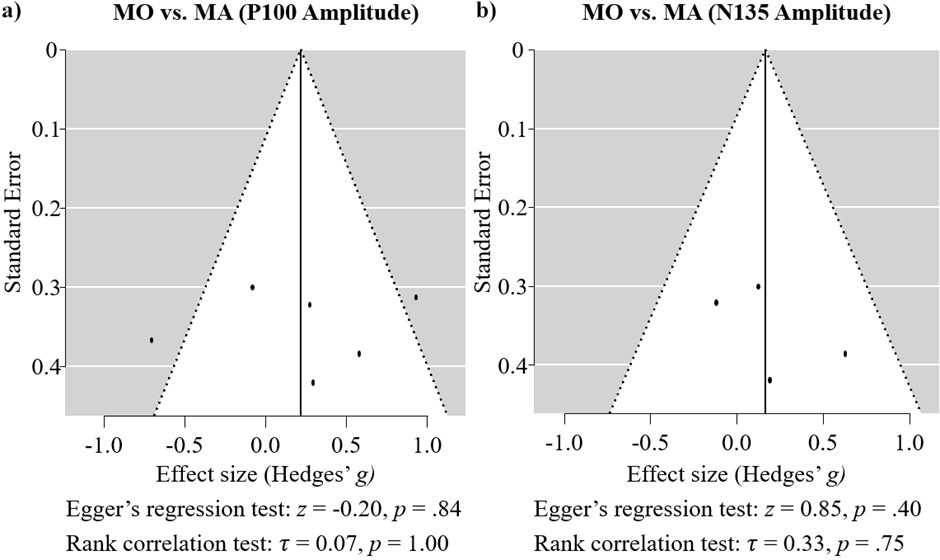

Supplement: Supplementary file 9 — Supplementary file9 (DOCX 91 KB) [file 11065_2022_9562_MOESM9_ESM.docx]

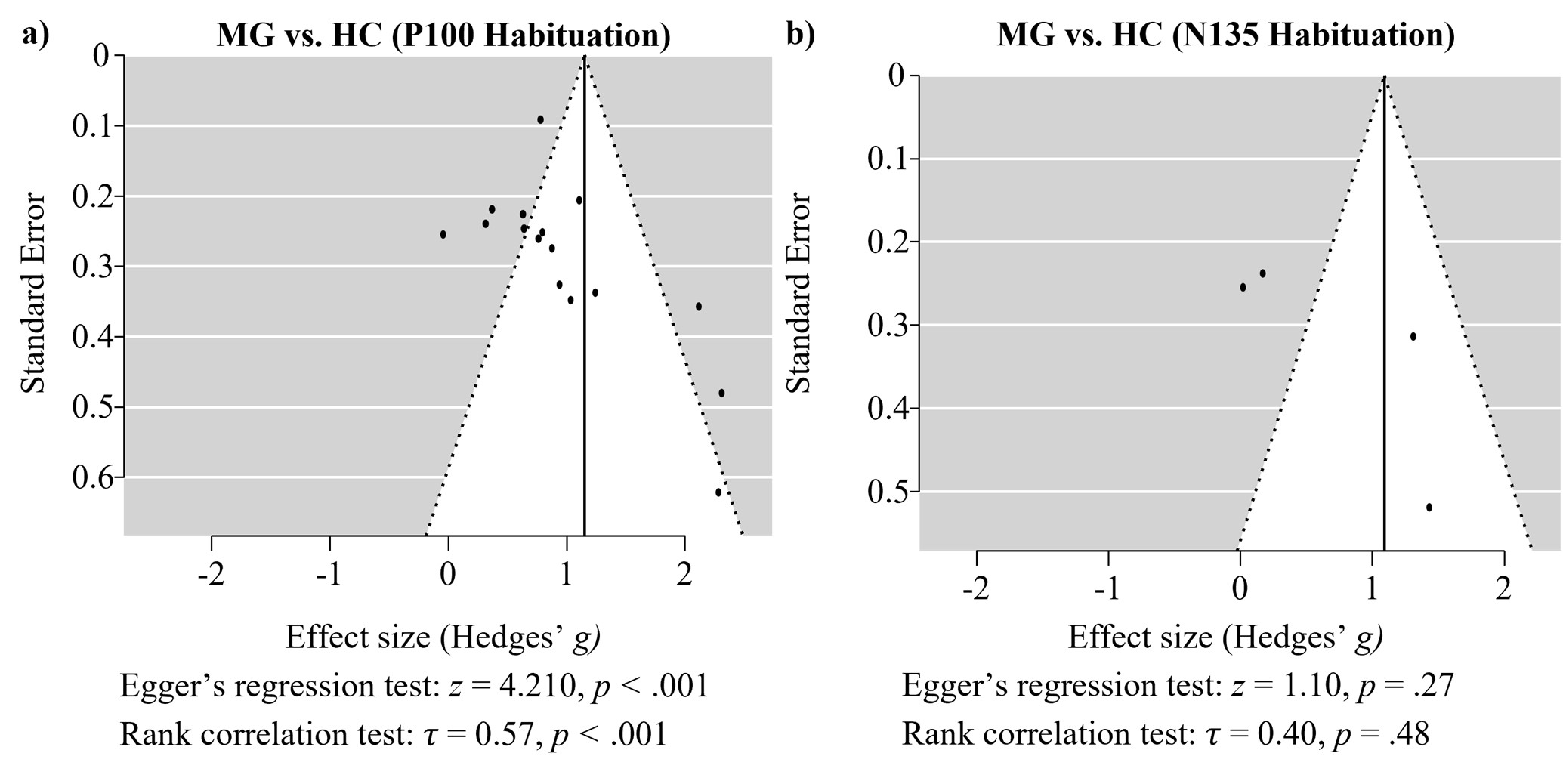

Supplement: Supplementary file 10 — Supplementary file10 (DOCX 215 KB) [file 11065_2022_9562_MOESM10_ESM.docx]

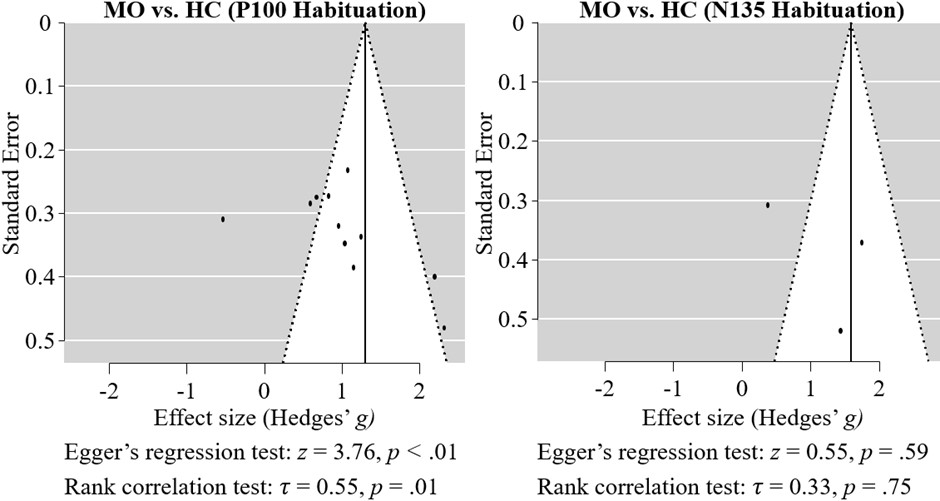

Supplement: Supplementary file 11 — Supplementary file11 (DOCX 87 KB) [file 11065_2022_9562_MOESM11_ESM.docx]

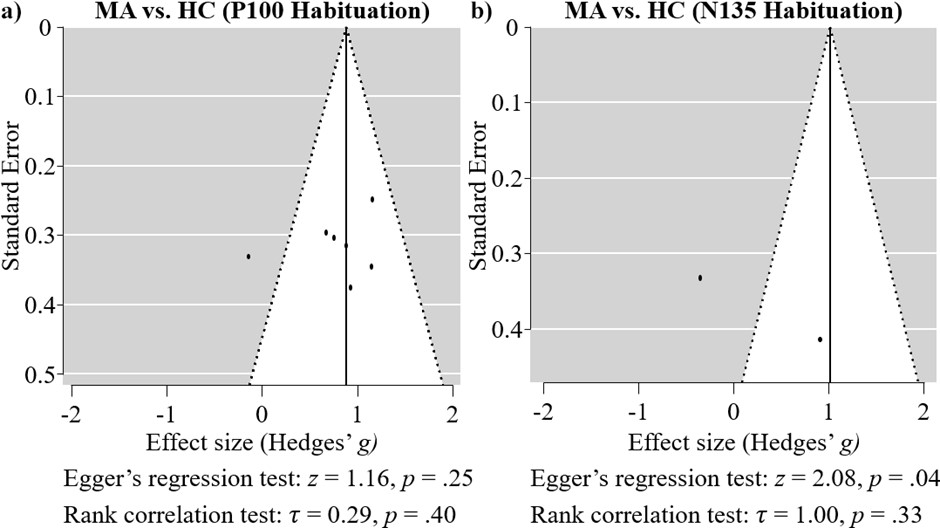

Supplement: Supplementary file 12 — Supplementary file12 (DOCX 93 KB) [file 11065_2022_9562_MOESM12_ESM.docx]

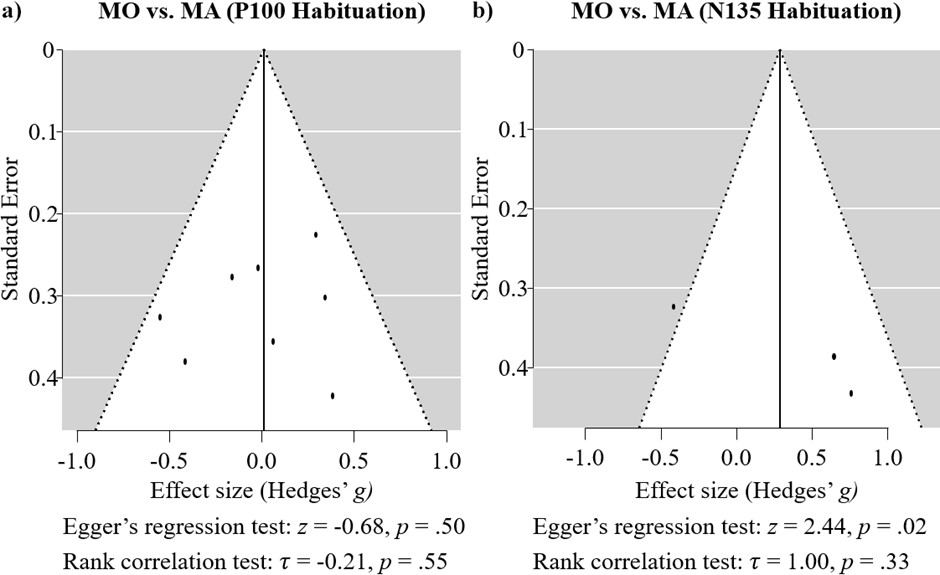

Supplement: Supplementary file 13 — Supplementary file13 (DOCX 94 KB) [file 11065_2022_9562_MOESM13_ESM.docx]
