# Supplementary material for: Visual Processing During the Interictal Period Between Migraines: A Meta-Analysis
Source: Neuropsychol Rev. 2022 Sep 17;33(4):765–82. doi: 10.1007/s11065-022-09562-3 (PMC10770263; doi:10.1007/s11065-022-09562-3)
Supplement: Supplementary file 16 — Supplementary file16 (PDF 107 KB) [file 11065_2022_9562_MOESM16_ESM.pdf]

Migraineurs (MG) vs Healthy Controls (HC)<br>Amplitude<br>P100 (*N* = 17)	N135 (*N* = 6)	Habituation<br>P100 (*N* = 17)	N135 (*N* = 5)
Afra et al., 1998	Afra et al., 1998	Afra et al., 1998	Afra et al., 1998
Afra et al., 2000b	Bohotin et al., 2002	Afra et al., 2000a	Bohotin et al., 2002
Ambrosini et al., 2015	Coppola et al., 2010a	Ambrosini et al., 2015	Coppola et al., 2010a
Bohotin et al., 2002	Coppola et al., 2013	Ambrosini et al., 2016a	Omland et al., 2013
Coppola et al., 2007	Omland et al., 2013	Ambrosini et al., 2016b	Omland et al., 2016
Coppola et al., 2010a	Omland et al., 2016	Bohotin et al., 2002	
Coppola et al., 2010b		Coppola et al., 2007	
Coppola et al., 2013		Coppola et al., 2010a	
Ince et al., 2017		Coppola et al., 2013	
Lisicki et al., 2017		Fumal et al., 2006	
Lisicki et al., 2018		Ince et al., 2017	
Logi et al., 2001		Judit et al., 2000	
Nguyen et al., 2012		Lisicki et al., 2017	
Nguyen et al., 2014		Omland et al., 2013	
Omland et al., 2013		Omland et al., 2016	
Omland et al., 2016		Ozkul & Bozlar, 2002	
Rauschel et al., 2015		Rauschel et al., 2015	

Migraineurs without aura (MO) vs HC			
Amplitude		Habituation	
P100 (*N* = 11)	N135 (*N* = 5)	P100 (*N* = 12)	N135 (*N* = 4)
Afra et al., 1998	Afra et al., 1998	Afra et al., 1998	Afra et al., 1998
Ambrosini et al., 2015	Bohotin et al., 2002	Afra et al., 2000a	Bohotin et al., 2002
Bohotin et al., 2002	Coppola et al., 2010a	Ambrosini et al., 2015	Coppola et al., 2010a
Coppola et al., 2007	Coppola et al., 2013	Ambrosini et al., 2016a	Omland et al., 2013
Coppola et al., 2010a	Omland et al., 2013	Bohotin et al., 2002	
Coppola et al., 2010b		Coppola et al., 2007	
Coppola et al., 2013		Coppola et al., 2010a	
Lisicki et al., 2017		Coppola et al., 2013	
Lisicki et al., 2018		Judit et al., 2000	
Nguyen et al., 2012		Lisicki et al., 2017	
Omland et al., 2013		Omland et al., 2013	
		Ozkul & Bozlar, 2002	

Migraineurs with Aura (MA) vs HC			
Amplitude		Habituation	
P100 (*N* = 7)	N135 (*N* = 4)	P100 (*N* = 8)	N135 (*N* = 3)
Afra et al., 1998	Afra et al., 1998	Afra et al., 1998	Afra et al., 1998
Afra et al., 2000b	Bohotin et al., 2002	Afra et al., 2000a	Bohotin et al., 2002
Bohotin et al., 2002	Coppola et al., 2013	Ambrosini et al., 2016a	Omland et al., 2013
Coppola et al., 2007	Omland et al., 2013	Bohotin et al., 2002	
Coppola et al., 2013		Coppola et al., 2007	
Nguyen et al., 2012		Coppola et al., 2013	
Omland et al., 2013		Omland et al., 2013	
		Ozkul & Bozlar, 2002	

MO vs MA<br>Amplitude<br>P100 (*N* = 6)	N135 (*N* = 4)	Habituation<br>P100 (*N* = 8)	N135 (*N* = 3)
Afra et al., 1998	Afra et al., 1998	Afra et al., 1998	Afra et al., 1998
Bohotin et al., 2002	Bohotin et al., 2002	Afra et al., 2000a	Bohotin et al., 2002
Coppola et al., 2007	Coppola et al., 2013	Ambrosini et al., 2016a	Omland et al., 2013
Coppola et al., 2013	Omland et al., 2013	Bohotin et al., 2002	
Nguyen et al., 2012		Coppola et al., 2007	
Omland et al., 2013		Coppola et al., 2013	
		Omland et al., 2013	
		Ozkul & Bozlar, 2002	
